# Beyond Deep Vein Thrombosis: Early Recognition of Phlegmasia Cerulea Dolens With Arterial Inflow Compromise and Concomitant Pulmonary Embolism

**DOI:** 10.7759/cureus.116580

**Published:** 2026-09-20

**Authors:** Humaid Sadiq, Bijy Abraham, Amani Alabdouli, Thiagarajan Jaiganesh

**Affiliations:** 1 Emergency Medicine, Tawam Hospital, Sheikh Tahnoon Bin Mohammed Medical City (STMC), Al Ain, ARE

**Keywords:** catheter-directed thrombolysis, deep vein thrombosis (dvt), iliofemoral deep vein thrombosis, mechanical thrombectomy (mt), phlegmasia cerulea dolens (pcd)

## Abstract

Phlegmasia cerulea dolens (PCD) is a rare and severe complication of extensive deep vein thrombosis (DVT) that can rapidly progress to limb ischemia, venous gangrene, and death. Early recognition and restoration of venous outflow are, therefore, critical.

We report the case of a 48-year-old man who presented with a three-day history of progressive left lower-limb swelling, pain, and bluish discoloration. Examination revealed tense swelling of the entire limb, delayed capillary refill, and a diminished dorsalis pedis pulse. Duplex ultrasonography demonstrated extensive left iliofemoral and popliteal DVT. Computed tomography (CT) angiography showed absent distal arterial opacification without primary arterial occlusion, consistent with arterial inflow compromise secondary to severe venous congestion. Despite the absence of cardiopulmonary symptoms or hemodynamic instability, CT pulmonary angiography demonstrated acute right lower-lobe segmental pulmonary emboli. The patient underwent therapeutic anticoagulation, inferior vena cava filter placement, and ultrasound-assisted catheter-directed thrombolysis, followed by mechanical thrombectomy and high-pressure venous angioplasty for persistent thrombus. Intravascular ultrasound confirmed satisfactory luminal expansion and excluded May-Thurner syndrome. Completion venography demonstrated restoration of brisk venous flow, with subsequent clinical improvement and preservation of limb viability.

This case highlights the importance of early recognition of PCD in patients presenting with a painful, swollen, cyanotic limb. Severe venous congestion in PCD can reduce distal arterial inflow even without primary arterial occlusion. Pulmonary embolism may also occur without respiratory symptoms or hemodynamic instability. Prompt anticoagulation, urgent multidisciplinary assessment, and timely escalation to endovascular thrombus removal may be critical for successful limb salvage.

## Introduction

Phlegmasia cerulea dolens (PCD) is a rare but severe form of deep vein thrombosis (DVT) caused by extensive obstruction of the major venous outflow of an extremity. It is typically characterized by the rapid development of severe limb swelling, pain, and cyanotic or violaceous discoloration. As venous pressure rises, marked interstitial edema may progressively impair tissue perfusion and compromise arterial inflow, potentially resulting in compartment syndrome, venous gangrene, limb loss, circulatory collapse, and death [1-3]. PCD most commonly involves the lower extremities and is frequently associated with extensive iliofemoral thrombosis. Reported predisposing factors include malignancy, thrombophilia, recent surgery or trauma, prolonged immobilization, pregnancy, and previous venous thromboembolism, although apparently unprovoked cases may also occur [1-3]. Early clinical recognition is essential, as prompt anticoagulation and, in selected patients with threatened limb viability, urgent endovascular thrombus-removal strategies may rapidly restore venous outflow and prevent irreversible ischemic injury [1,3,4].

We report this case because it illustrates several clinically important and potentially overlapping challenges in the emergency evaluation of an acutely swollen, cyanotic limb. The patient presented with extensive iliofemoral DVT and evolving PCD, with distal arterial inflow compromise in the absence of primary arterial occlusion, as well as clinically silent pulmonary embolism despite hemodynamic stability. The case highlights the importance of early recognition of limb-threatening PCD from bedside findings, distinguishing venous-induced arterial compromise from primary arterial occlusion, and maintaining vigilance for concomitant pulmonary embolism even in the absence of cardiopulmonary symptoms. Prompt escalation to multimodal endovascular therapy was critical when limb viability was threatened.

## Case presentation

A 48-year-old male truck driver presented to the emergency department (ED) with a three-day history of progressive swelling of the left lower extremity. The symptoms were preceded by bilateral lower back pain, which had resolved spontaneously before the onset of the limb swelling. The swelling progressively worsened and became associated with moderate, diffuse pain extending from the left hip to the foot, prompting him to seek emergency medical attention.

His medical history was significant for class I obesity and active cigarette smoking. Shortly before symptom onset, he had been driving continuously for approximately two to three hours. He denied recent prolonged immobilization, long-distance travel, trauma, surgery, hospitalization, malignancy, or previous venous thromboembolism. There was no known personal or family history of thrombophilia. He denied fever, chest pain, shortness of breath, exertional dyspnea, orthopnea, paroxysmal nocturnal dyspnea, palpitations, dizziness, cough, or hemoptysis. He also denied any recent skin or soft tissue infection involving the affected limb.

On arrival, he was alert and oriented, appearing mildly distressed because of left leg pain. He was hemodynamically stable, with a blood pressure of 128/95 mmHg, a heart rate of 79 beats/minute, a respiratory rate of 20 breaths/minute, a temperature of 37.0°C, and an oxygen saturation of 98% on room air. Examination of the left lower limb demonstrated marked, tense swelling extending from the hip to the foot, with erythematous to bluish discoloration compared with the contralateral limb (Figure 1). Mild pitting edema was present over the left foot, and capillary refill was prolonged to approximately 3-4 seconds. The left dorsalis pedis pulse was difficult to palpate and appeared diminished compared with the right side, although this was thought to be partly related to the significant edema. Despite these findings, the limb remained warm, with preserved motor function and sensation. Examination of the right lower limb was unremarkable. Cardiovascular and respiratory examinations were normal, while abdominal examination revealed mild epigastric tenderness without guarding or distension.

**Figure 1:**
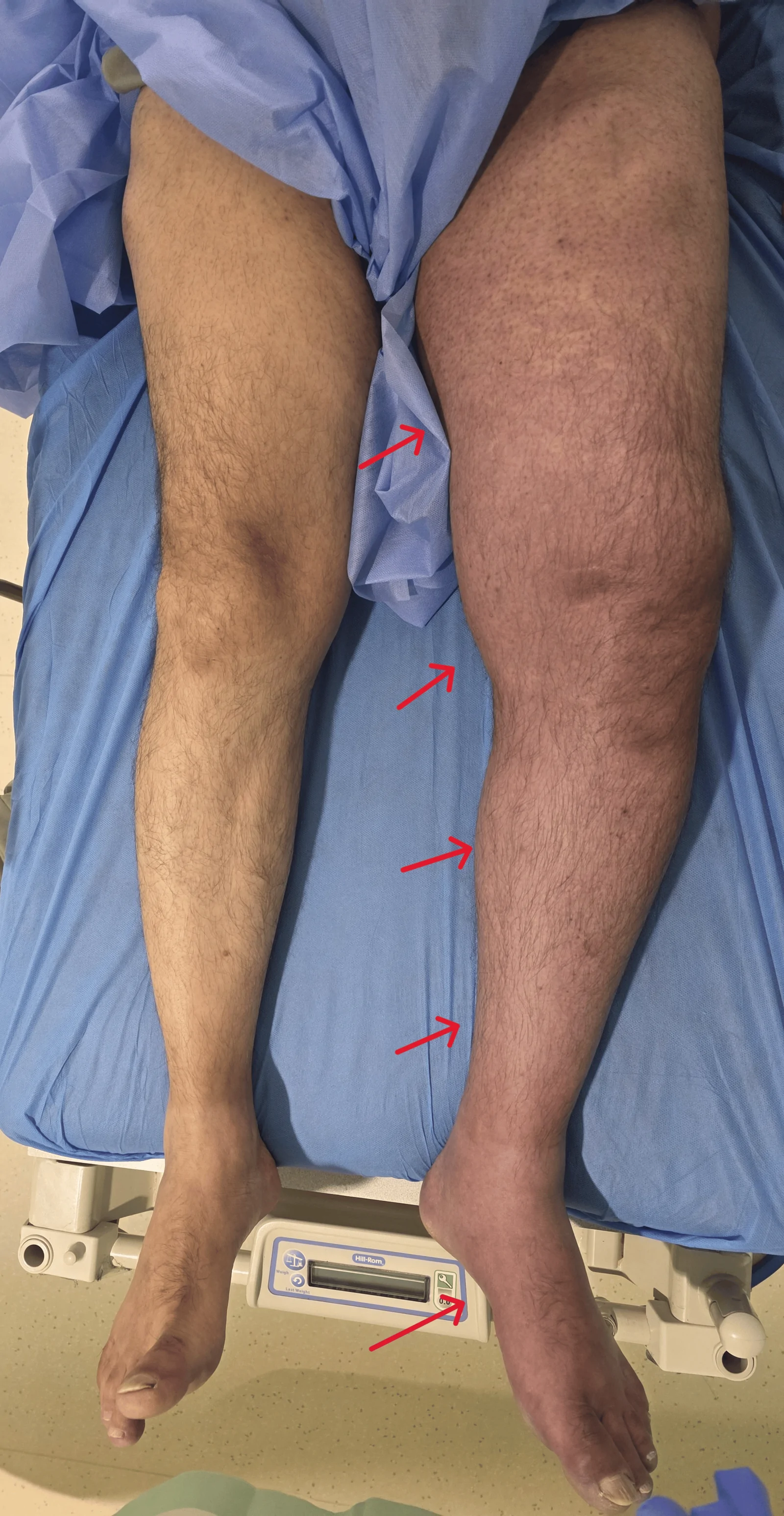
Clinical appearance of phlegmasia cerulea dolens (PCD). Marked unilateral swelling and erythematous-to-bluish discoloration of the left lower extremity compared with the right lower extremity, consistent with PCD. Red arrows highlight areas of discoloration and swelling. Consent was obtained from the patient for publication of this clinical photograph.

Investigations

Initial laboratory investigations demonstrated leukocytosis with neutrophilia, an elevated C-reactive protein level of 55.4 mg/L, and a markedly elevated D-dimer level. Renal function, serum electrolytes, coagulation profile, and venous blood gas parameters were within normal limits, including a lactate level of 1.1 mmol/L. High-sensitivity troponin I and N-terminal pro-B-type natriuretic peptide (NT-proBNP) were not elevated (Table 1). Electrocardiography (ECG) demonstrated normal sinus rhythm (Figure 2), and chest radiography showed no acute cardiopulmonary abnormality (Figure 3).

**Table 1:** Initial blood investigations in the emergency department.

Test	Result	Reference range
White blood cells (WBC)	11.3	4.5-11.0 x10^9^/L
Red blood cells (RBC)	4.94	4.20-5.60 x10^12^/L
Platelet	291	140-400 x10^9^/L
Absolute neutrophils	8.67	1.80-7.70 x10^9^/L
C-reactive protein	55.5	<=5.0 mg/L
D-Dimer	10.410	0.129-0.523 mg/L
Prothrombin time (PT)	10.9	9.9-11.8 sec(s)
International normalized ratio (INR)	1.00	0.87-1.15
Sodium (Na)	138	136–145 mmol/L
Potassium (K)	4.1	3.5–5.1 mmol/L
Chloride (Cl)	112	98–107 mmol/L
Creatinine	83.7	53.0-114.9 micromol/L
Urea	7.40	3.20-7.40 mmol/L
Estimated glomerular filtration rate (eGFR)	95	>=60 mL/min
Troponin-I	<5.10	<=34.20 ng/L
N-terminal pro-B-type natriuretic peptide (NT-proBNP)	43.1	0.0-125.0 ng/L
Lactate	1.1	0.50-2.20 mmol/L

**Figure 2:**
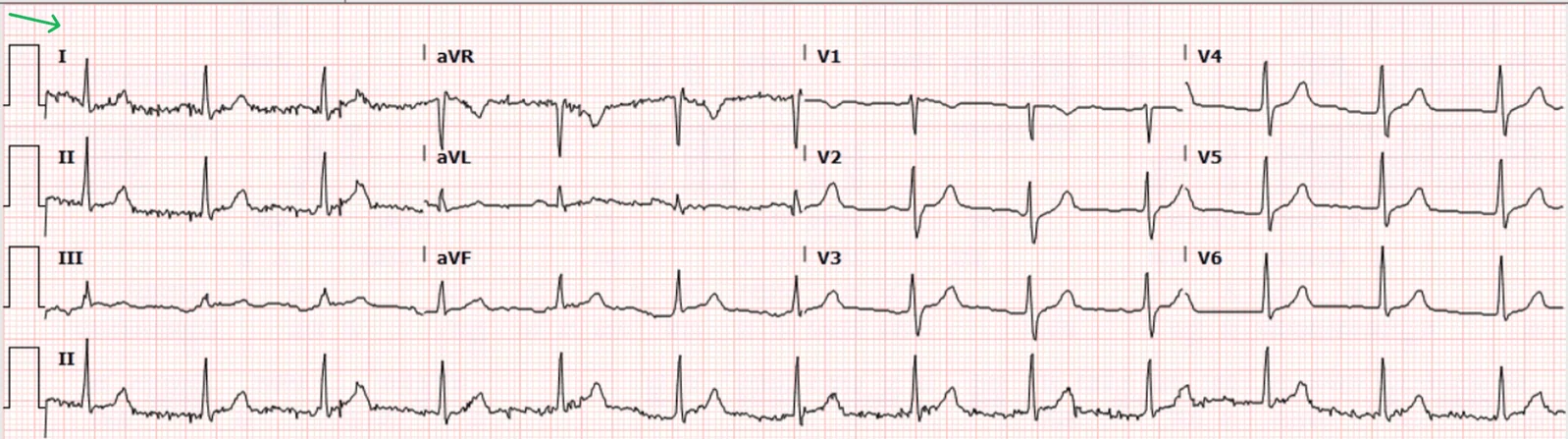
Normal electrocardiogram (as indicated by the green arrow).

**Figure 3:**
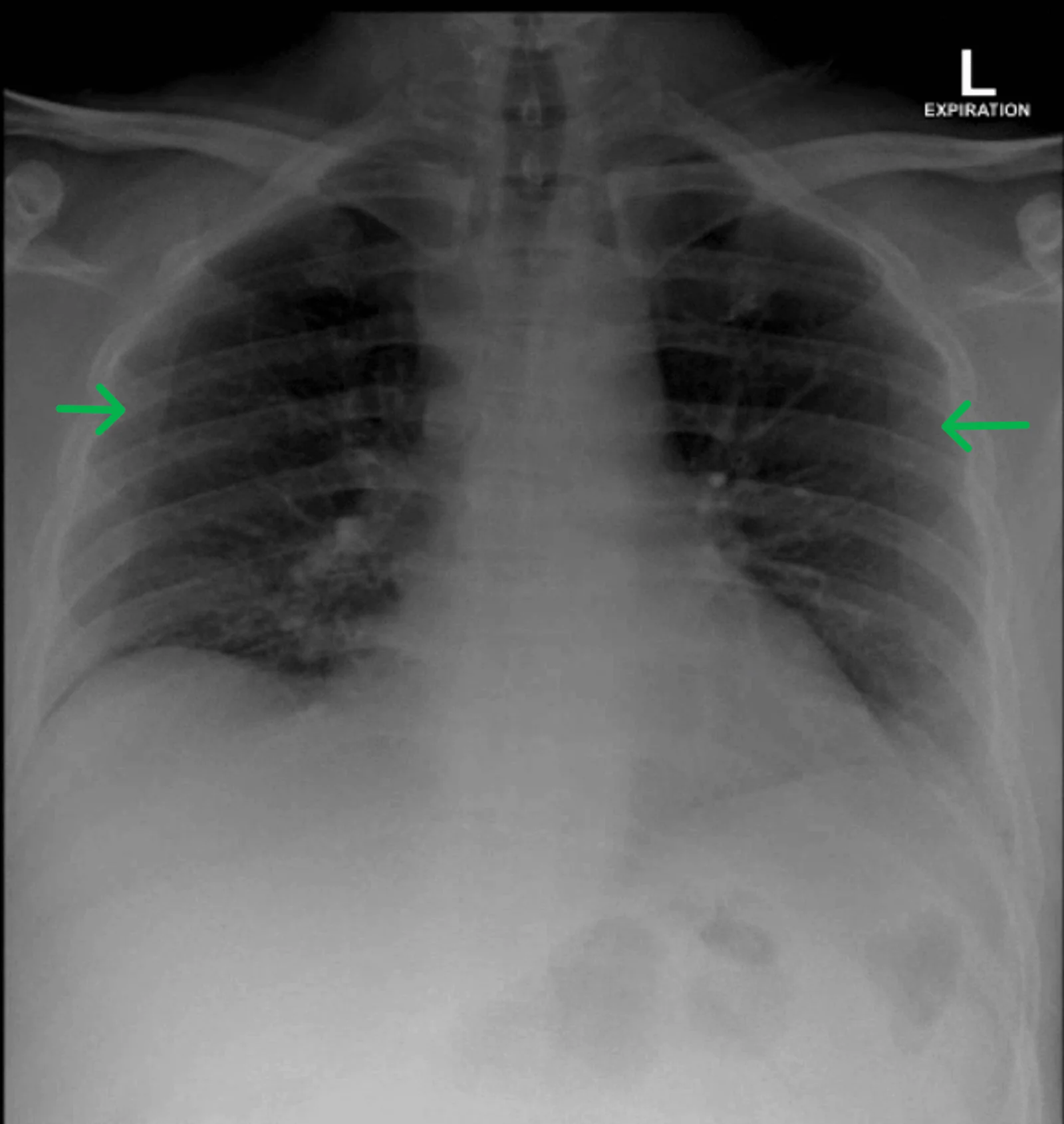
Chest X-ray showing no acute cardiopulmonary abnormality (as indicated by the green arrows).

Given the marked unilateral swelling, bluish discoloration, delayed capillary refill, and reduced distal pulse, there was concern for extensive proximal venous thrombosis with evolving limb compromise. Urgent duplex ultrasonography demonstrated acute thrombosis involving the left external iliac, common femoral, femoral, and popliteal veins, with no thrombosis identified in the contralateral limb (Figures 4-8).

**Figure 4:**
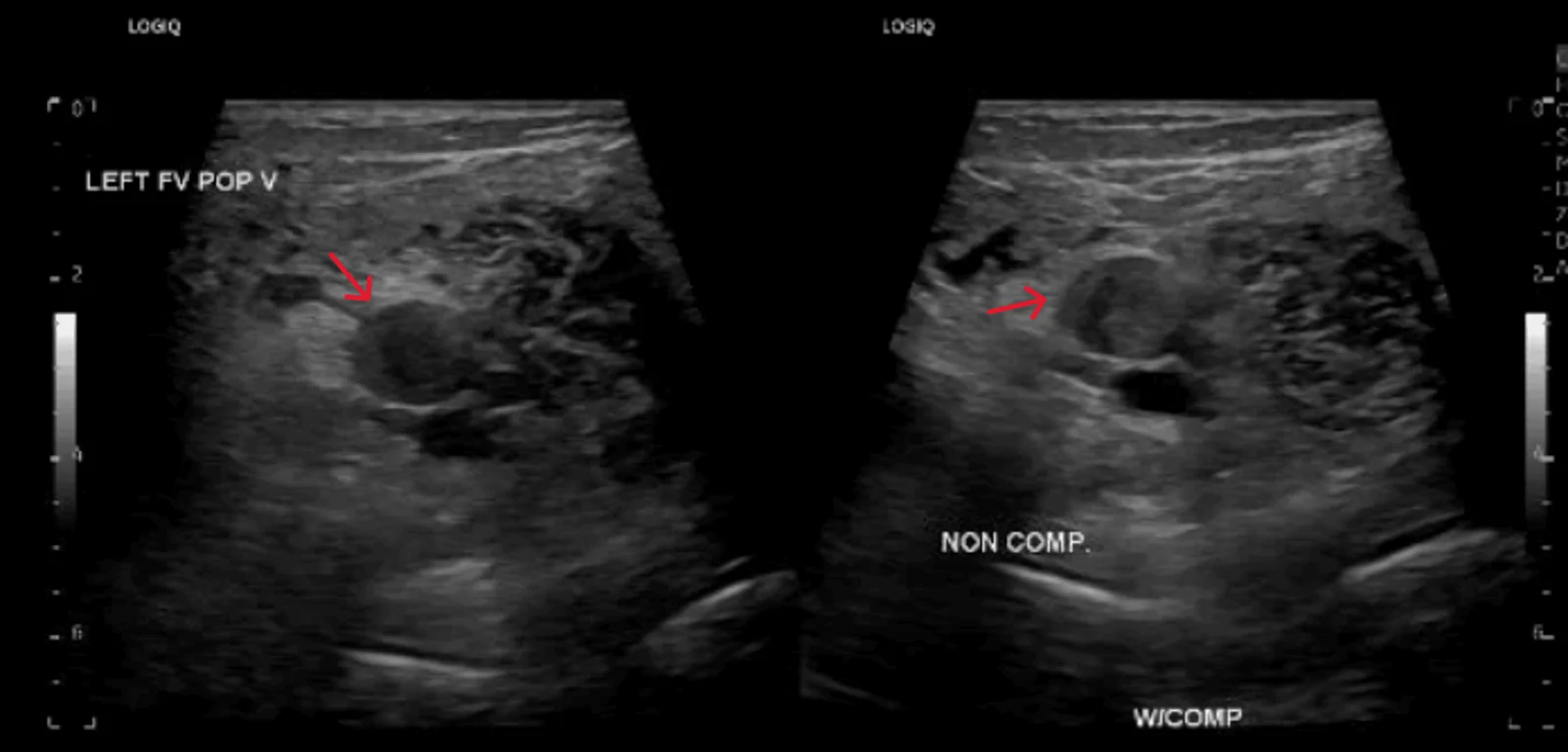
Ultrasound of the left popliteal vein demonstrating a non-compressible vein (as indicated by red arrows).

**Figure 5:**
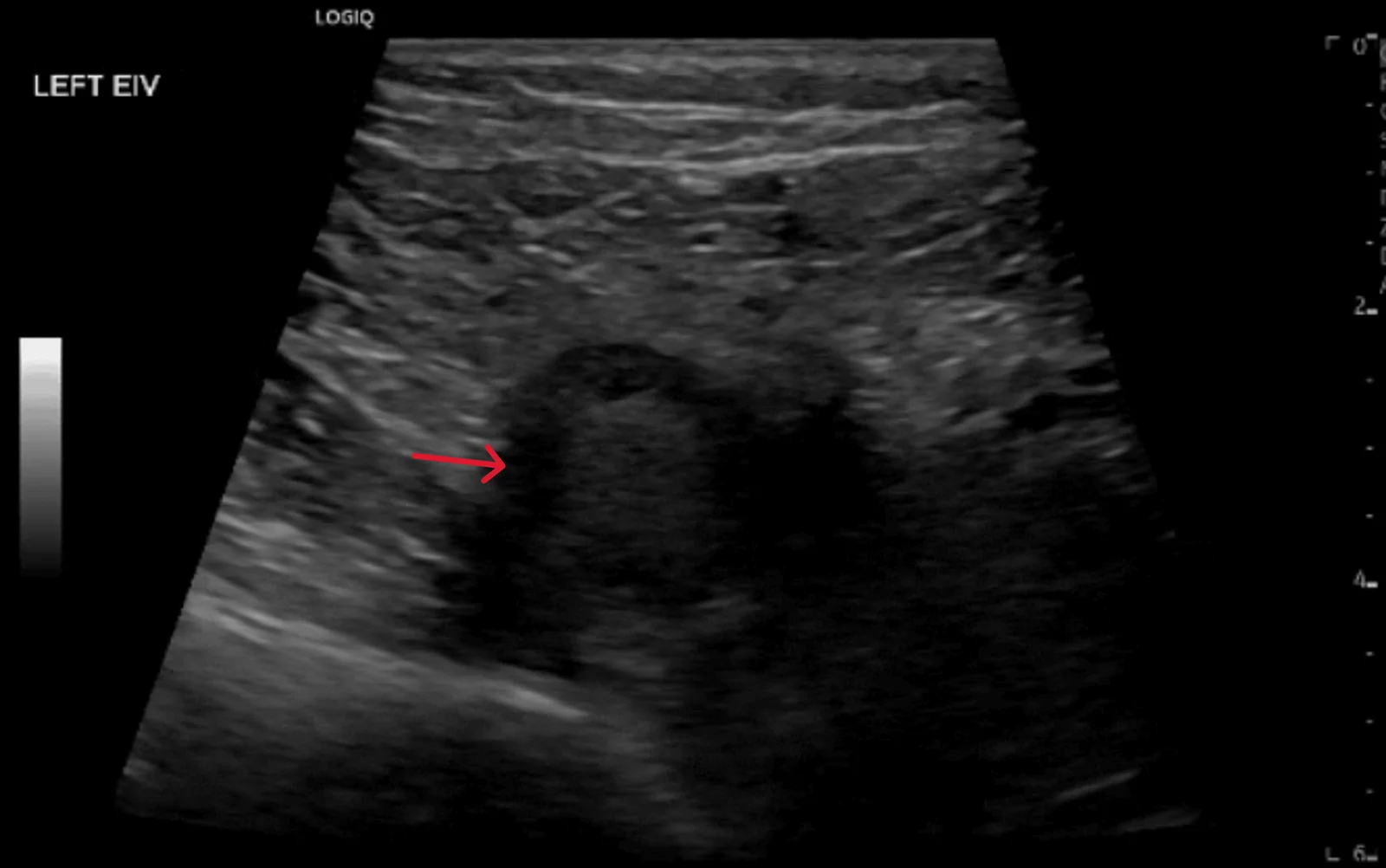
Ultrasound of the left external iliac vein demonstrating a non-compressible vein (as indicated by red arrows).

**Figure 6:**
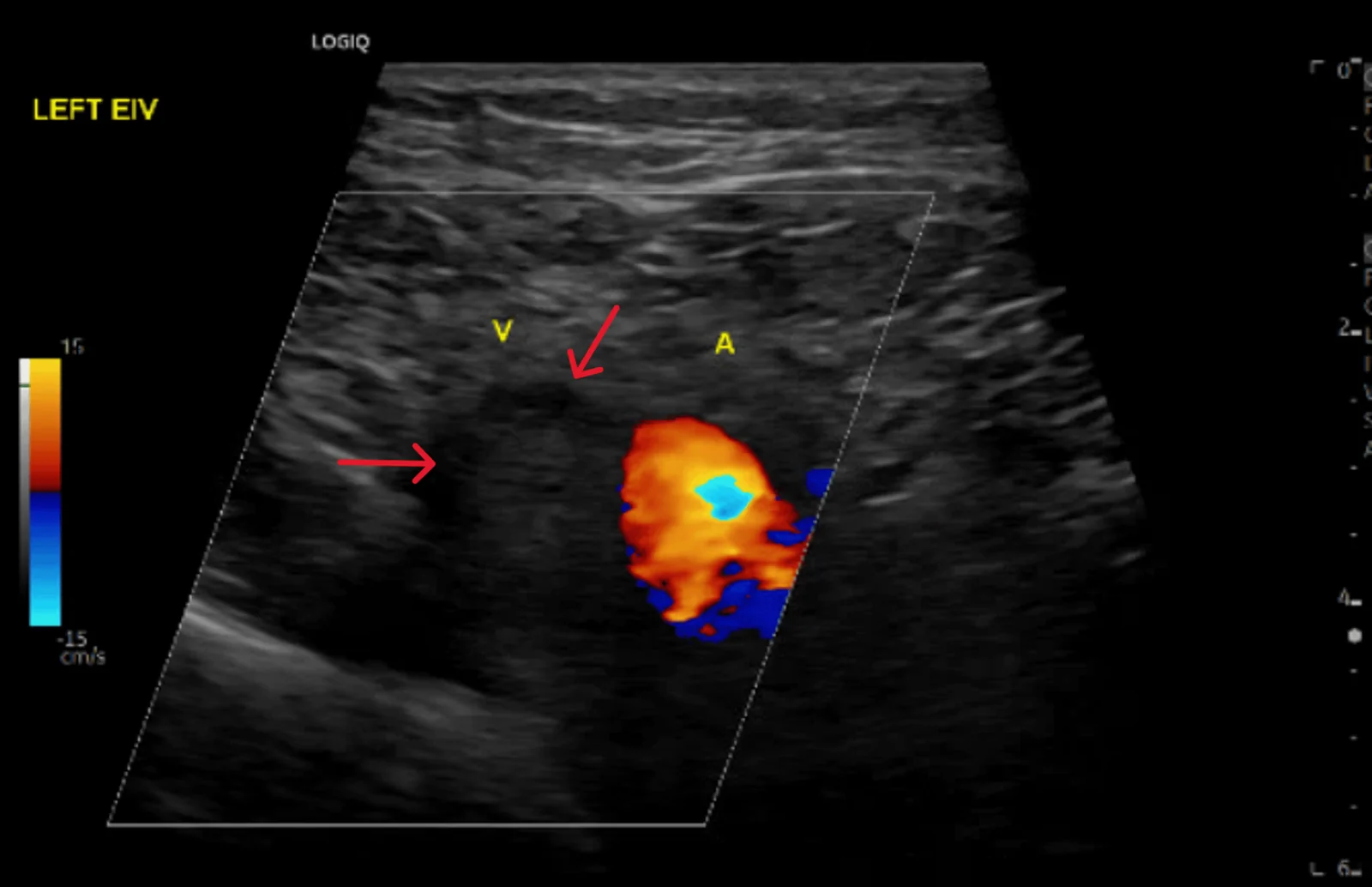
Ultrasound color Doppler of the left external iliac vein demonstrating absence of blood flow (as indicated by red arrows).

**Figure 7:**
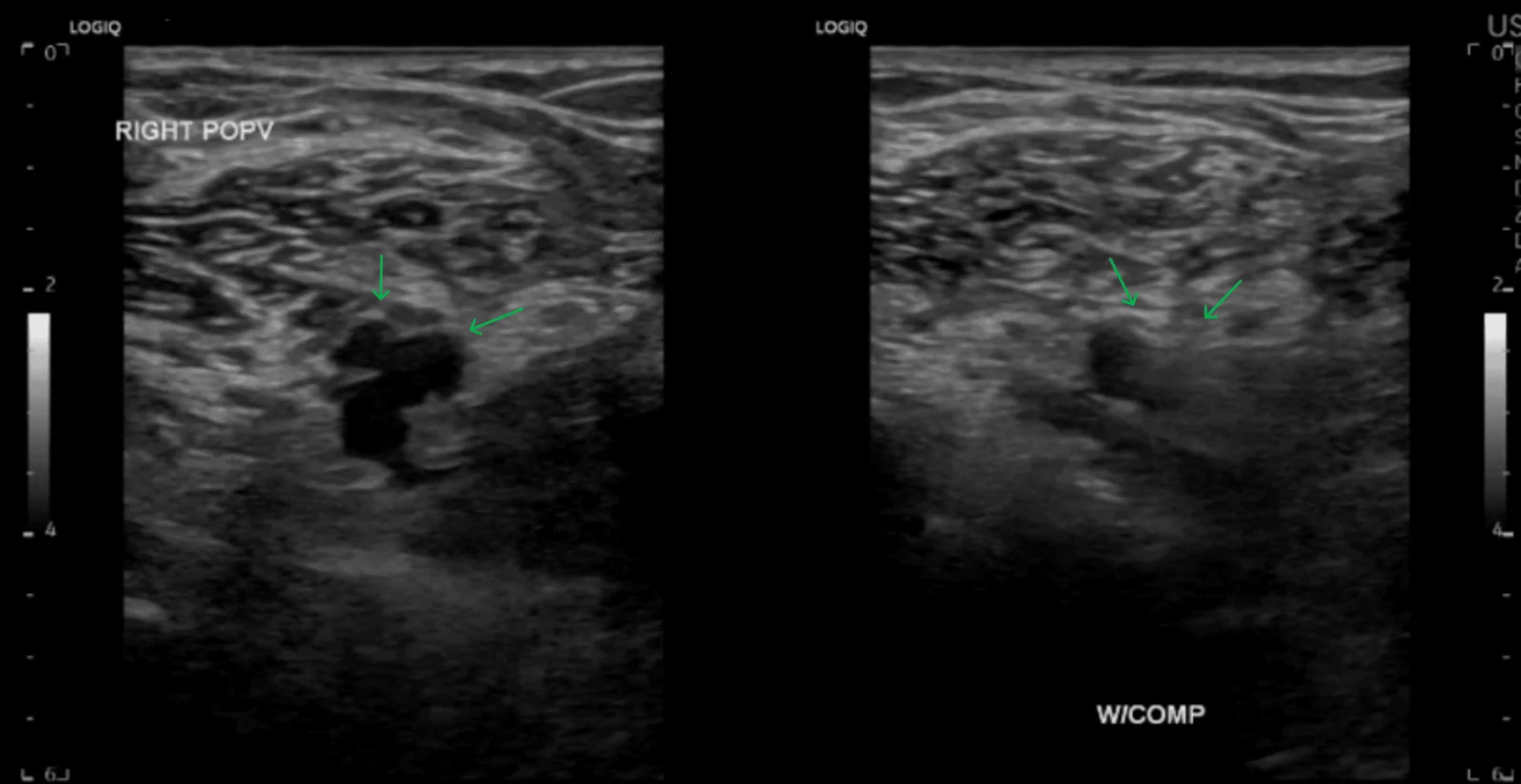
Ultrasound of the right popliteal vein demonstrating the compressibility of the vein (as indicated by green arrows).

**Figure 8:**
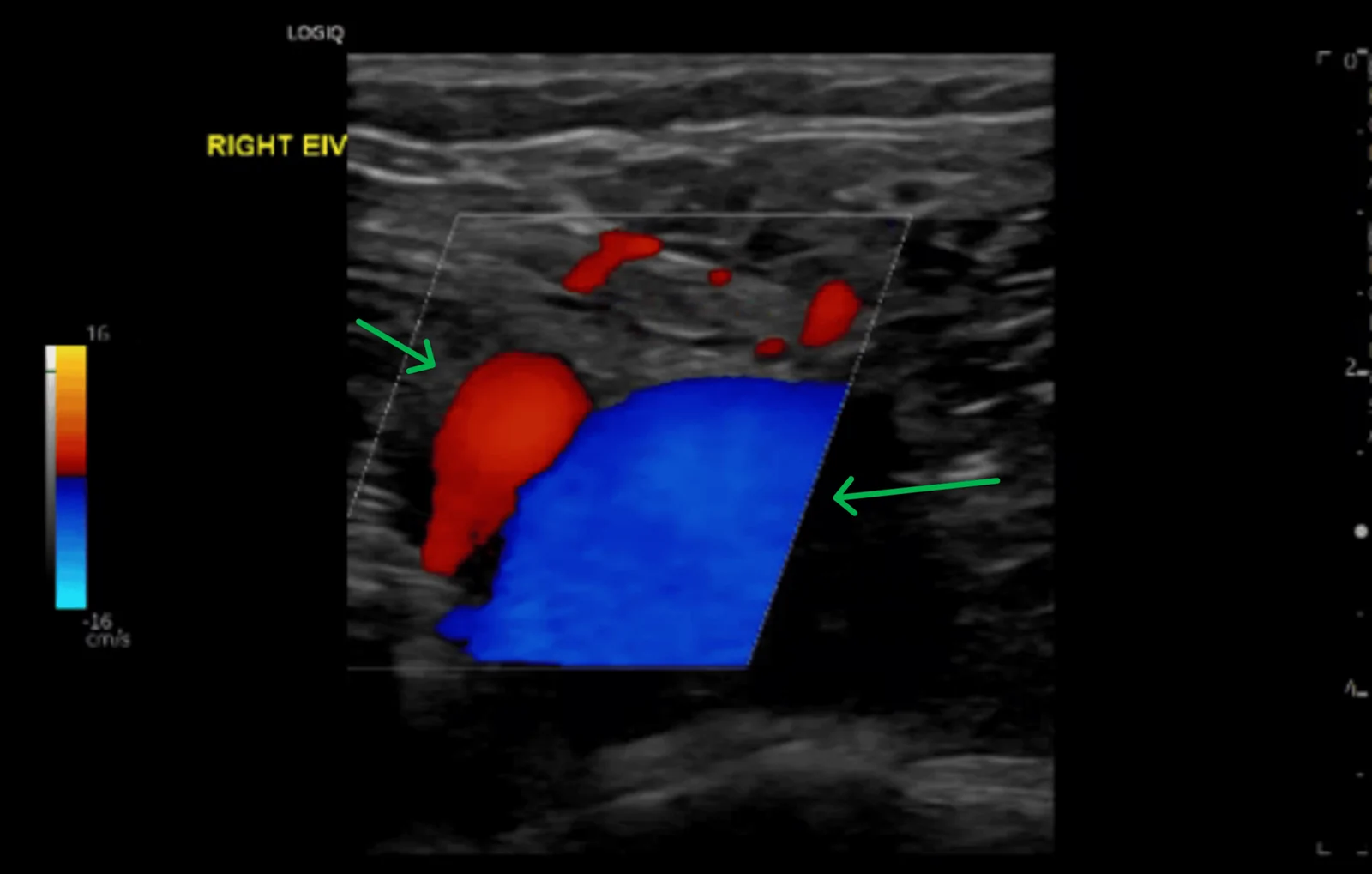
Ultrasound color Doppler of the right external iliac vein demonstrating the presence of blood flow (as indicated by green arrows).

Because of the extensive thrombus burden and clinical concern for PCD, computed tomography (CT) angiography of the abdomen, pelvis, and lower extremities was performed (Figure 9). This confirmed extensive acute left iliofemoral and popliteal deep venous thrombosis with surrounding inflammatory changes consistent with thrombophlebitis. Importantly, there was absent arterial opacification within the distal left lower extremity despite no evidence of a primary arterial thrombus or occlusion, raising concern for impaired arterial inflow secondary to severe venous congestion.

**Figure 9:**
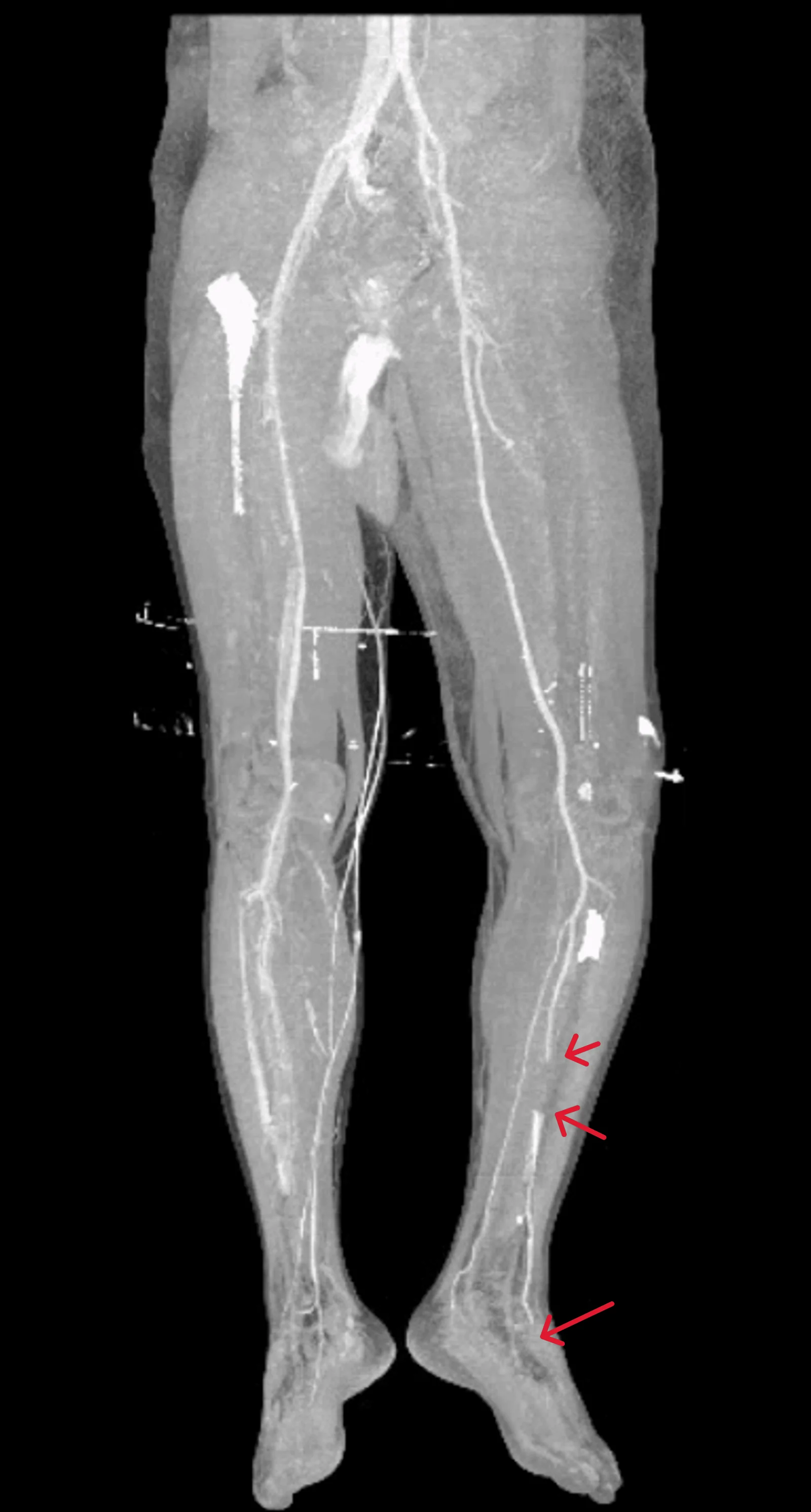
Computed tomography (CT) angiography of the lower extremities demonstrating absent arterial opacification within the distal left lower extremity.

Although the patient had no respiratory symptoms and remained hemodynamically stable, CT pulmonary angiography was performed because of the extensive proximal thrombus burden. This demonstrated acute pulmonary emboli involving segmental branches of the right lower lobe, without pulmonary infarction or imaging evidence of right ventricular strain (Figure 10). The overall clinical and radiologic findings were therefore consistent with PCD secondary to extensive left iliofemoral DVT, complicated by concomitant acute pulmonary embolism.

**Figure 10:**
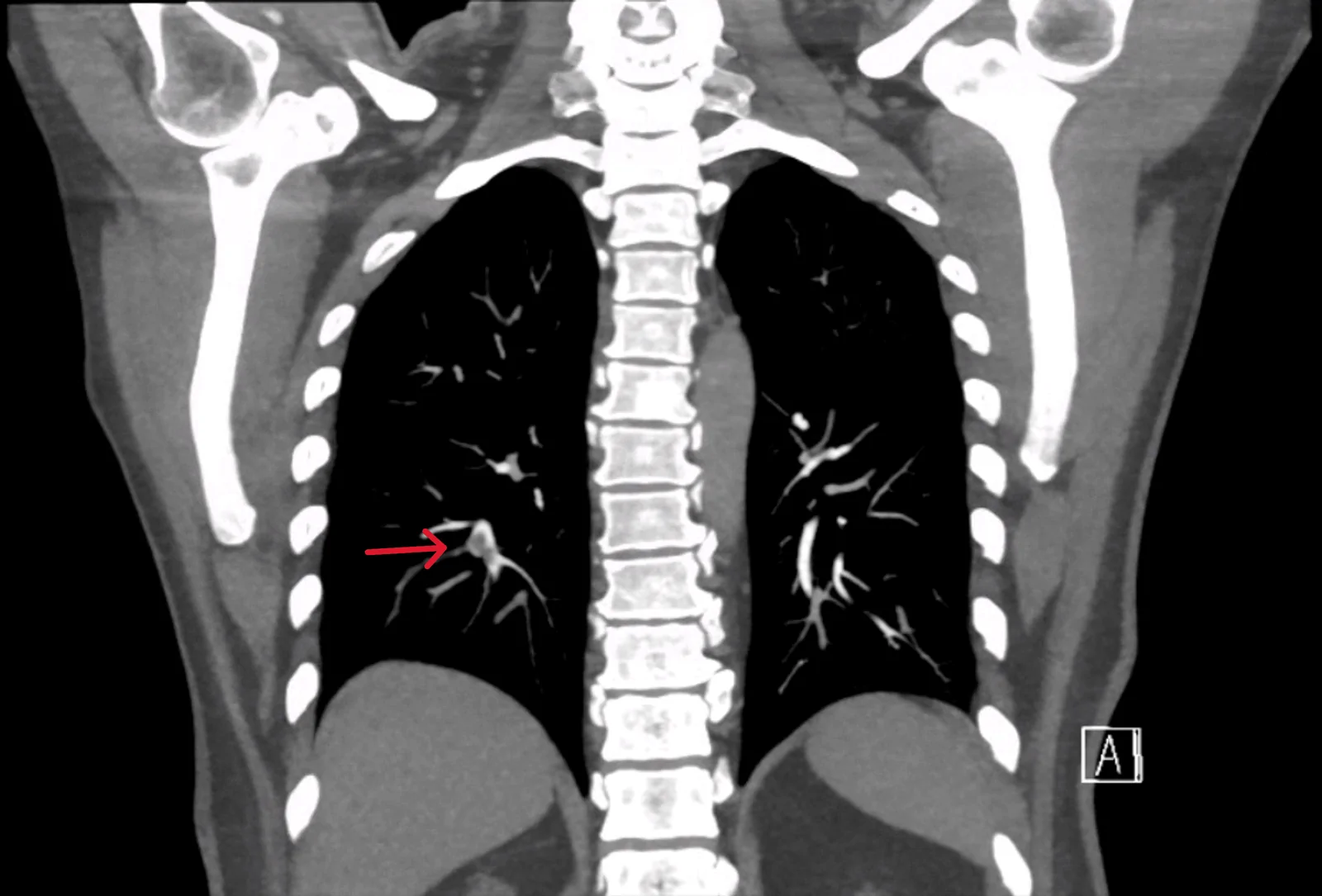
Computed tomography (CT) pulmonary angiography demonstrating acute pulmonary emboli involving segmental branches of the right lower lobe.

Treatment

Therapeutic anticoagulation with low-molecular-weight heparin was initiated promptly following confirmation of extensive iliofemoral DVT on duplex ultrasonography. Because of the clinical concern for threatened limb viability, the patient underwent urgent endovascular intervention. An inferior vena cava filter was inserted, followed by ultrasound-assisted catheter-directed thrombolysis using an EKOS™ Endovascular System (Boston Scientific Corporation, Marlborough, MA, USA) catheter positioned within the left femoral venous system, with continuous recombinant tissue plasminogen activator infusion. He was subsequently admitted to the intensive care unit for close postprocedural monitoring.

A second-look venography performed the following day demonstrated persistent subtotal occlusion extending to the left common iliac vein with a substantial residual thrombus burden. Mechanical thrombectomy using the AngioJet system was therefore performed, followed by high-pressure balloon angioplasty of the left common iliac, external iliac, and femoral veins to optimize venous outflow. Intravascular ultrasound confirmed excellent luminal expansion, with no significant residual or floating thrombus. Completion venography demonstrated complete restoration of brisk inline venous flow from the left femoral vein through the iliac venous system into a widely patent inferior vena cava.

Outcome and follow-up

The patient remained hemodynamically stable throughout his admission without bleeding or other procedural complications. He demonstrated progressive improvement in pain, swelling, and limb discoloration following intervention, with preservation of limb viability and no evidence of venous gangrene.

Anticoagulation was continued with therapeutic low-molecular-weight heparin (enoxaparin 1 mg/kg twice daily) and subsequently transitioned to oral edoxaban after completion of five days of parenteral anticoagulation. He was discharged home after five days with oral anticoagulation and scheduled follow-up with the vascular surgery and pulmonology teams.

## Discussion

PCD is a rare but severe manifestation of DVT, characterized by extensive venous outflow obstruction leading to marked limb swelling, pain, and cyanotic discoloration. In advanced cases, massive venous hypertension and interstitial edema may compromise arterial inflow, resulting in tissue ischemia, venous gangrene, compartment syndrome, limb loss, and death [1-3]. Early recognition is therefore essential, as progression from extensive DVT to irreversible limb-threatening ischemia can be rapid.

The clinical findings in our patient were consistent with evolving PCD. He had progressive tense swelling of the entire left lower limb, erythematous-to-bluish discoloration, prolonged capillary refill, and a reduced dorsalis pedis pulse. Importantly, the limb remained warm, with preserved motor function and sensation, suggesting that irreversible limb ischemia had not yet developed. Duplex ultrasonography confirmed extensive thrombosis involving the external iliac, common femoral, femoral, and popliteal veins.

A particularly important feature of this case was the finding of absent distal arterial opacification on CT angiography despite the absence of a primary arterial occlusion. This phenomenon can occur in severe PCD when markedly elevated venous and interstitial pressures reduce the arteriovenous pressure gradient and impair arterial inflow. A recent case report described PCD with secondary arterial inflow compromise due to extensive iliofemoral venous obstruction, successfully treated with pharmacomechanical thrombectomy [5]. This supports the concept that diminished distal arterial opacification in PCD may result from severe venous hypertension rather than an intrinsic arterial thrombotic event.

Another clinically important feature was the presence of concomitant pulmonary embolism despite the absence of respiratory symptoms or hemodynamic instability. CT pulmonary angiography demonstrated segmental right lower-lobe pulmonary emboli without evidence of pulmonary infarction or right ventricular strain. Pulmonary embolism is a recognized complication of PCD and has been associated with adverse outcomes [6,7]. In our patient, the presence of clinically silent pulmonary embolism despite a stable cardiopulmonary presentation highlights that extensive venous thromboembolism may coexist with an initially reassuring cardiopulmonary assessment.

PCD should therefore be regarded as a vascular emergency rather than simply an extensive DVT. Initial management includes prompt therapeutic anticoagulation, limb elevation, analgesia, and urgent vascular or interventional assessment [4]. However, anticoagulation alone may be insufficient when there is evidence of threatened limb viability, as it does not rapidly relieve the mechanical obstruction caused by extensive thrombus. Chang and Chang reported successful treatment of PCD using intravenous heparin followed by AngioJet mechanical thrombectomy, angioplasty, and catheter-directed thrombolysis, with restoration of venous flow and an uncomplicated recovery [1]. Their report highlights the potential role of aggressive thrombus-removal strategies when extensive venous obstruction threatens limb viability.

The patient reported two to three hours of continuous driving shortly before symptom onset. Although prolonged immobility is a recognized risk factor for venous thromboembolism, this relatively limited duration cannot be established as a causal factor, and the event was considered apparently unprovoked in the absence of other major provoking factors.

Catheter-directed thrombolysis has also been successfully used in selected patients with PCD. A previous case report described two patients with PCD associated with gynaecological malignancy who were successfully treated with catheter-directed venous thrombolysis, highlighting the potential role of targeted thrombolytic therapy in achieving rapid recanalization and limb salvage [8].

In our patient, the initial catheter-directed thrombolytic approach was followed by a second-look venogram, which demonstrated substantial residual thrombus and persistent venous obstruction. This necessitated additional mechanical thrombectomy and high-pressure balloon angioplasty, with intravascular ultrasound confirming satisfactory luminal expansion and exclusion of significant residual thrombus and May-Thurner syndrome. The final venogram demonstrated restoration of brisk inline venous flow, confirming successful technical and anatomical restoration of venous outflow and coinciding with rapid clinical improvement. This case therefore illustrates the potential value of a staged, multimodal endovascular approach when extensive thrombus persists despite initial thrombolytic therapy.

In contrast, delayed or unsuccessful restoration of venous drainage can result in catastrophic complications. PCD may progress to venous gangrene, compartment syndrome, circulatory shock, multiorgan failure, and death [3,9]. A reported case of right lower-extremity PCD complicated by compartment syndrome progressed to circulatory shock and cardiac arrest; hemodynamic instability subsequently precluded definitive thrombolysis or thrombectomy, and the patient died [9]. Such cases reinforce the importance of recognizing PCD early, before irreversible limb or systemic complications develop. The principal learning point is that early recognition and rapid restoration of venous outflow are critical in PCD. In this limb-threatening condition, time is limb.

## Conclusions

PCD is a rare but potentially devastating complication of extensive DVTs and should be regarded as a limb- and life-threatening vascular emergency. Recognition of the characteristic combination of severe limb swelling, pain, and cyanotic discoloration is essential, particularly when delayed capillary refill or diminished distal pulses suggests evolving compromise of arterial inflow. Our case demonstrates that distal arterial inflow compromise in advanced PCD can occur as a consequence of severe venous congestion even in the absence of primary arterial occlusion. Early duplex ultrasonography followed by appropriate cross-sectional imaging allowed rapid characterization of the thrombus burden and extent of limb compromise.

Urgent anticoagulation, combined with ultrasound-assisted catheter-directed thrombolysis, mechanical thrombectomy, and venous angioplasty, successfully restored venous outflow and preserved limb viability. Anticoagulation alone may be insufficient when limb viability is threatened, and timely escalation to endovascular thrombus removal may be required to achieve venous decompression and limb salvage. Conversely, progression to compartment syndrome, venous gangrene, shock, or multiorgan failure may substantially limit therapeutic options and result in amputation or death.
